# Supplementary material for: Long-range atmospheric transport of microplastics across the southern hemisphere
Source: Nat Commun. 2023 Nov 30;14:7898. doi: 10.1038/s41467-023-43695-0 (PMC10689495; doi:10.1038/s41467-023-43695-0)
Supplement: Supplementary file 1 — Supplementary Information [file 41467_2023_43695_MOESM1_ESM.docx]

**Supplementary Information for**

**Long-range Atmospheric Transport of Microplastics across the Southern Hemisphere**

**Qiqing Chen**^1^**, Guitao Shi^2,1^*, Laura E. Revell^3^, Jun Zhang^4,5^, Chencheng Zuo**^1^**, Danhe Wang^2^, Eric Le Ru^6^, Guangmei Wu^2^, Denise M. Mitrano^7^**

^1^ State Key Laboratory of Estuarine and Coastal Research, East China Normal University, Shanghai 200241, China

^2^ Key Laboratory of Geographic Information Science (Ministry of Education), School of Geographic Sciences, East China Normal University, Shanghai 200241, China

^3^ School of Physical and Chemical Sciences, University of Canterbury, Christchurch 8140, New Zealand

^4^ Department of Physics, New York University, NY, NY 10003, USA

^5^ NYU-ECNU Physics and Mathematics Research Institutes, New York University Shanghai, Shanghai 200062, China

^6^ The MacDiarmid Institute for Advanced Materials and Nanotechnology, School of Chemical and Physical Sciences, Victoria University of Wellington, Wellington 6140, New Zealand

^7^ Department of Environmental Systems Science, ETH Zurich, Zurich 8092, Switzerland

**Table of Contents**

**Figures S1-S18**

**Tables S1-S5**

**Texts S1-S4**

**Supplementary Figures**


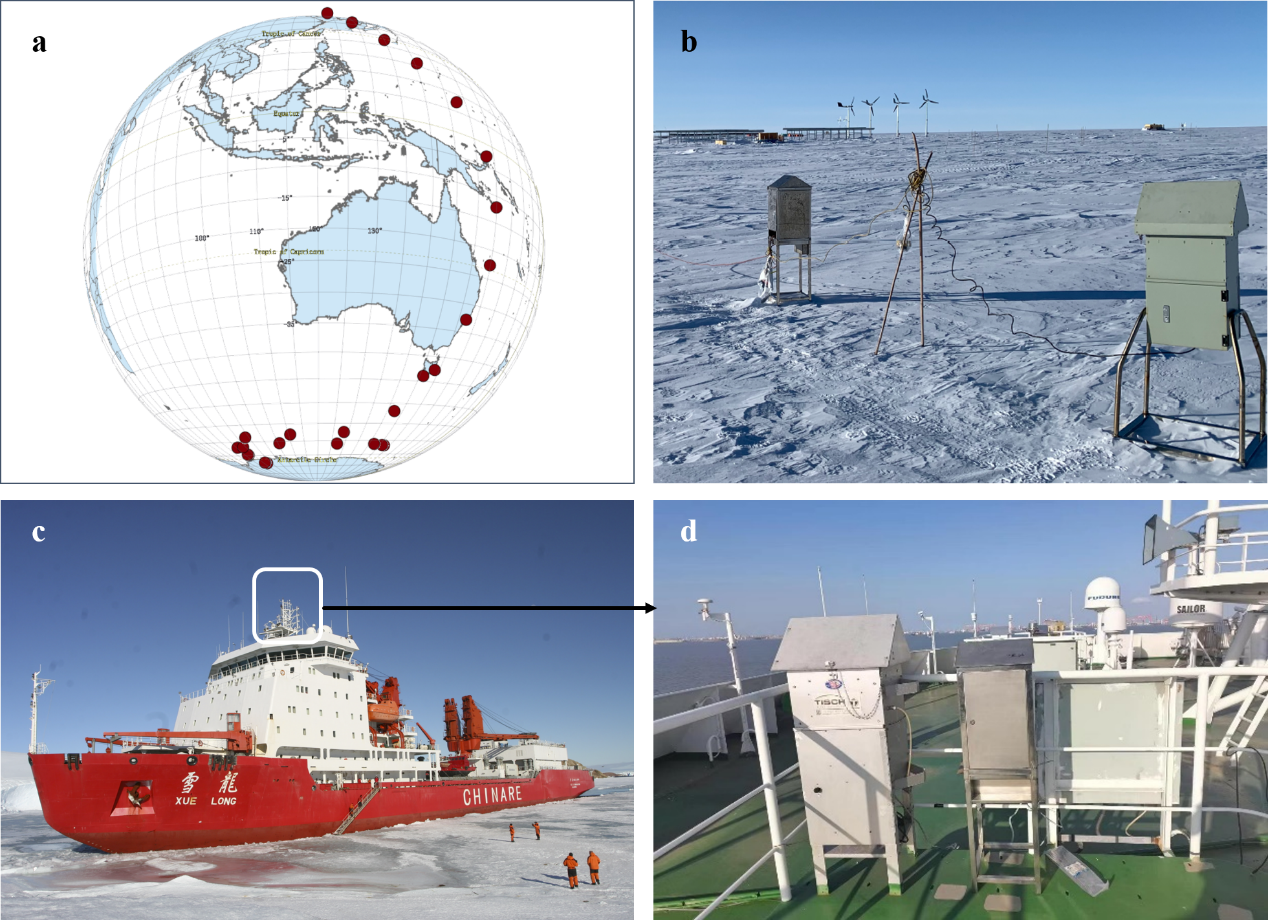


**Figure S1** Sampling sites in the marine atmospheric boundary layer from mid-Northern Hemisphere to Antarctica on the top deck of the RV/Xuelong (a, c, and d), and two atmosphere samples were collected in inland Antarctica, ~520 km from the coast (b). Panel a was made with the aid of ArcGIS 10.2 (ESRI Co, Redlands, USA).


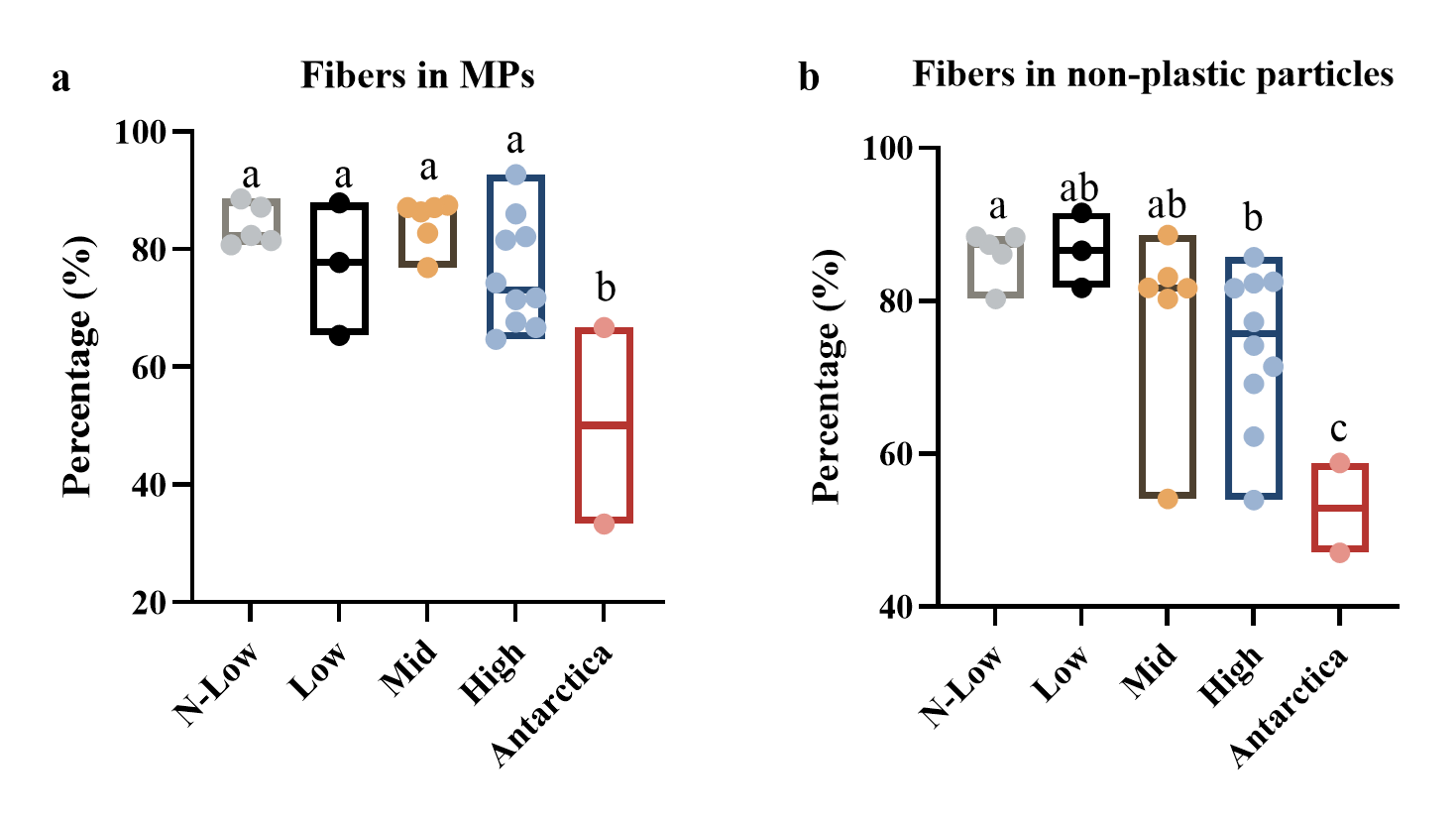


**Figure S2** Fiber composition percentages in samples. (a) Microplastics (MPs) fibers as a percentage of all MPs along the expedition transects; (b) Non-plastic fibers as percentages of all non-plastic particles along the expedition transects. N-low, low, mid, and high latitudes represent the regions of about 0-30^o^N, 0-30^o^S, 30-60^o^S, and 60-70^o^S along the cruise path; Antarctica: inland Antarctica. Different letters represent significant differences according to ANOVA analysis (*p*<0.05). Lines that cross the boxes are median values.


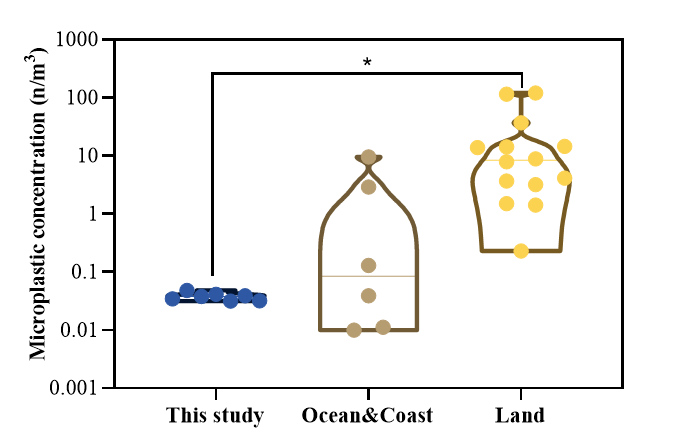


**Figure S3** Comparison of atmospheric microplastics (MPs) concentrations between previous studies and this study, with further detailed information in Table S3. * indicates a statistical difference between the two groups according to independent *t* tests (*p*<0.05). In reporting the results for this study, we calculated and plotted seven average values which correspond to latitudes of 0-15°N, 15-30°N, 0-15°S, 15-30°S, 30-45°S, 45-60°S, and 60-75°S, which are presented in the graph as one data point. Lines that cross the violin plots are median values.


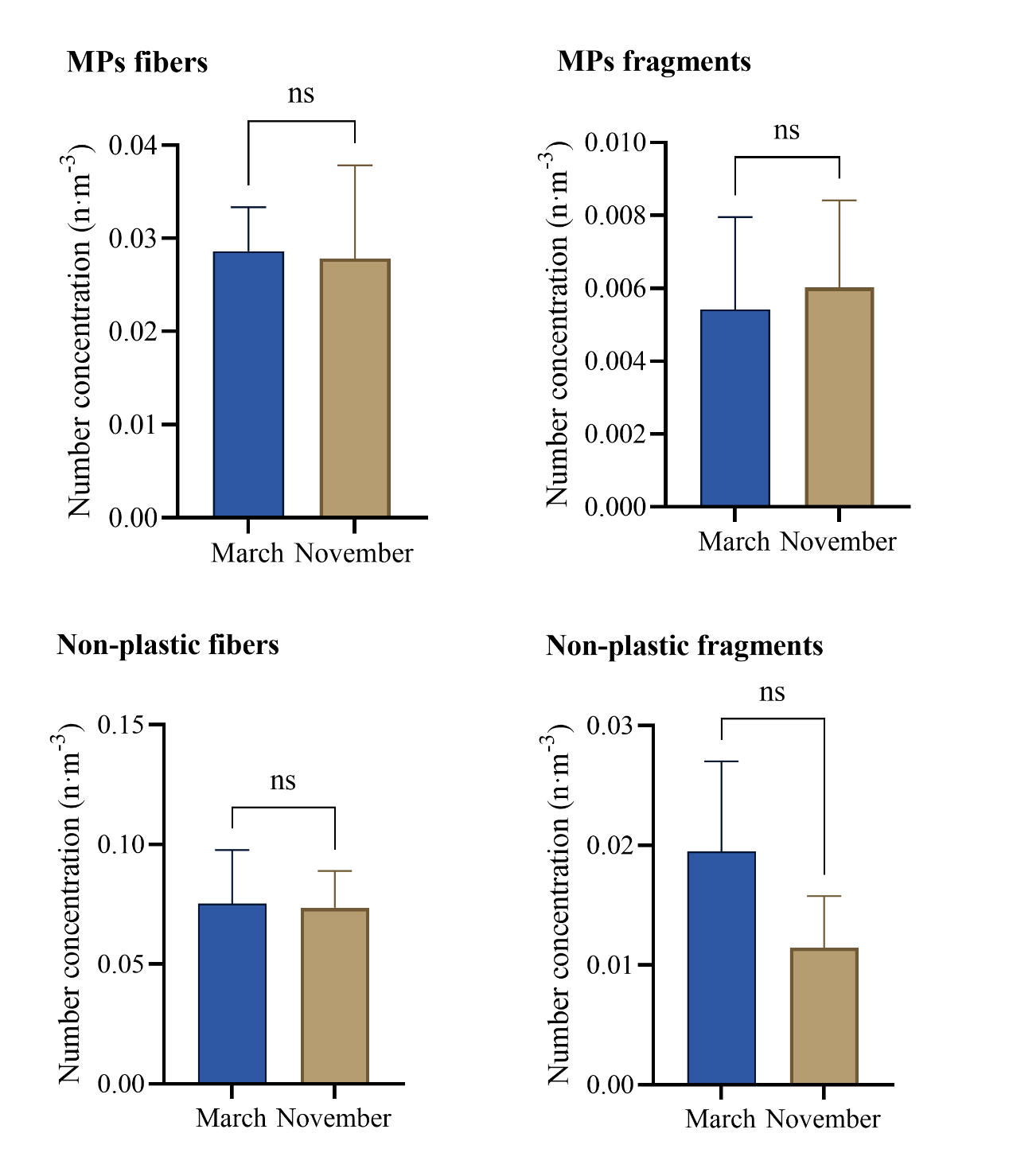


**Figure S4** Seasonal factors have limited influence on microplastics (MPs) and non-plastic particles number concentrations. A comparison between sampling locations A16-A21 (March, austral autumn) and A13-A15,22 (November, austral spring) within the latitudes of 60°S- 65°S. ns: indicates no statistically significant differences between the two groups (*p*>0.05) according to *t*-tests. Error bars represent one standard deviation.

**
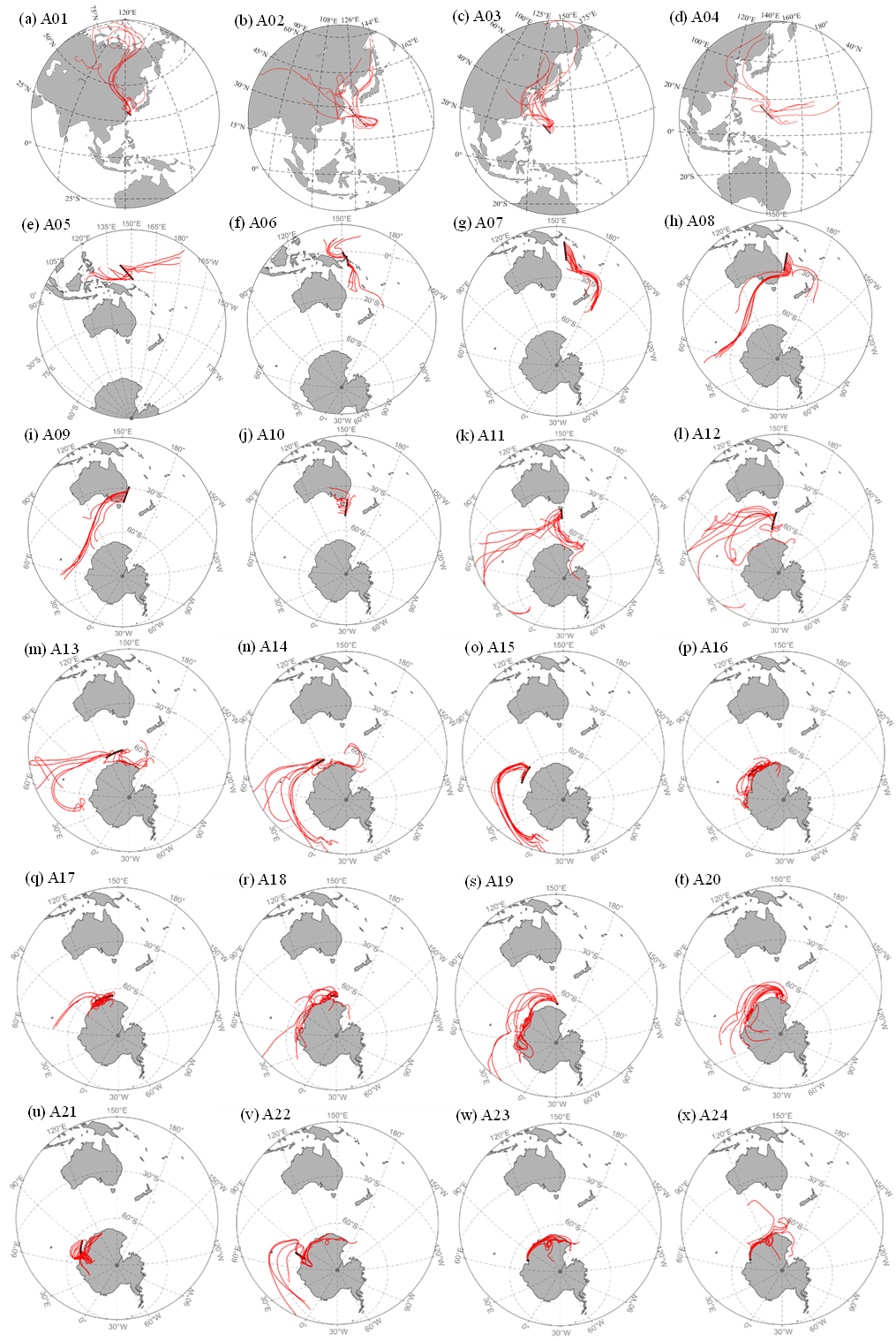
Figure S5** Backward trajectories of all samples collected in marine boundary layer. Detailed information of samples shown in each individual panel is provided in Table S1. This figure was made with the aid of HYSPLIT model, http://www.arl.noaa.gov/HYSPLIT.php. For more details refer to the main text.

**Figure S6** Number concentration of sealant tar microplastics along the cruise path. N-low, low, mid, and high latitudes represent the regions of about 0-30^o^N, 0-30^o^S, 30-60^o^S, and 60-70^o^S along the cruise path. * represents significant difference between N-Low and High (*p*<0.05). Error bars represent one standard deviation.

**Figure S7** Specific polymer composition of microplastics fibers at each latitude interval. PE: polyethylene, PET: polyethylene terephthalate, PVC: polyvinyl chloride, PP: polypropylene, PMMA: poly (methyl methacrylate), PVP: polyvinyl propionate, PS: polystyrene. Others include Covinax 81, poly (butyl acrylate), melamine formaldehyde resin, poly (ethylacrylate: acrylamide), and unspecified alkyd and olefin, etc.


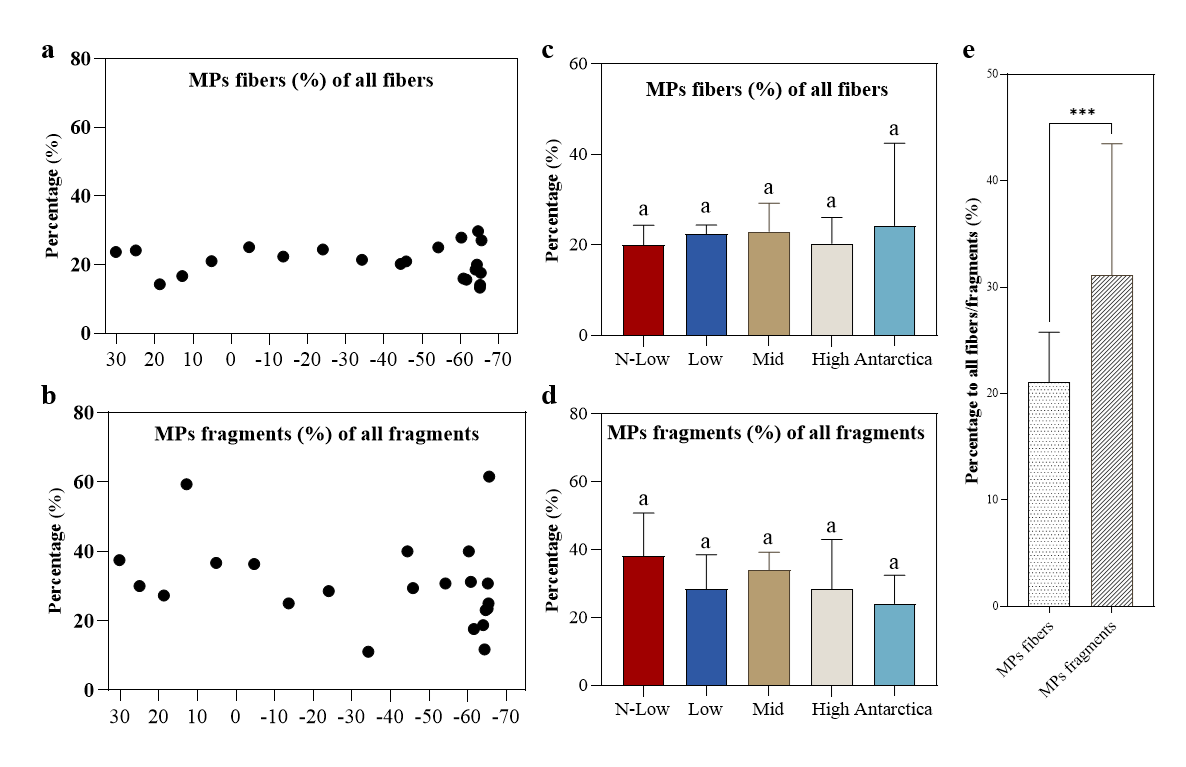


**Figure S8** The percentages of microplastics (MPs) in all fibers and fragments along the transect: (a) proportion of MPs fibers along the cruise path; (b) proportion of MPs fibers at different latitudes; (c) proportion of MPs fibers along the cruise path; (d) proportion of MPs fragments at different latitudes; (e) comparison of MPs proportions between fibers and fragments along the cruise path. N-low, low, mid, and high latitudes represent the regions of approximately 0-30^o^N, 0-30^o^S, 30-60^o^S, and 60-70^o^ along the cruise path, respectively. Antarctica indicates the two samples collected in inland Antarctica. The same letter of a above columns suggests there are no significant differences among groups. *** represents significant difference between MP fibers and MP fragments (*p*<0.001). Error bars represent one standard deviation.

**Figure S9** Polymer composition of microplastics (MPs) fragments at each latitude interval. S indicates south hemisphere; N indicates north hemisphere. PE: polyethylene, PUR: polyurethane, PS: polystyrene, PP: polypropylene, PS: polystyrene. Others include polybutyl acrylate, poly(vinyl acetate), polyacrylamide, and unspecified alkyd and olefin, etc.


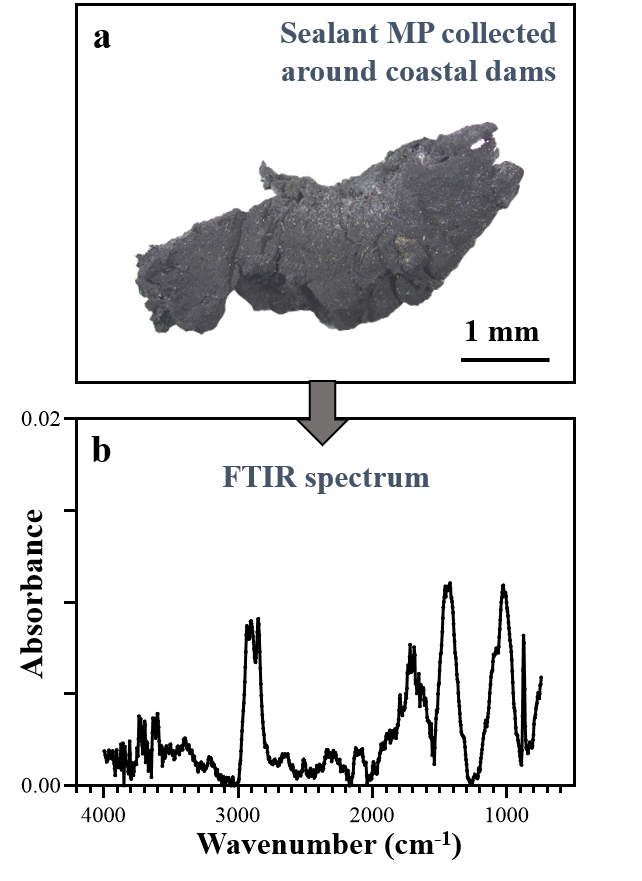


**Figure S10** Composition identification of dam sealant tar particles. (a) a typical sealant particle collected around the coastal dam along the East China Sea coast (31.0^o^N, 121.9^o^E); (b) the micro-FTIR spectrum of the sealant particle.

**Figure S11** Color distribution patterns of microplastics (MPs) fibers and fragments at different latitudes. S indicates south hemisphere; N indicates north hemisphere.


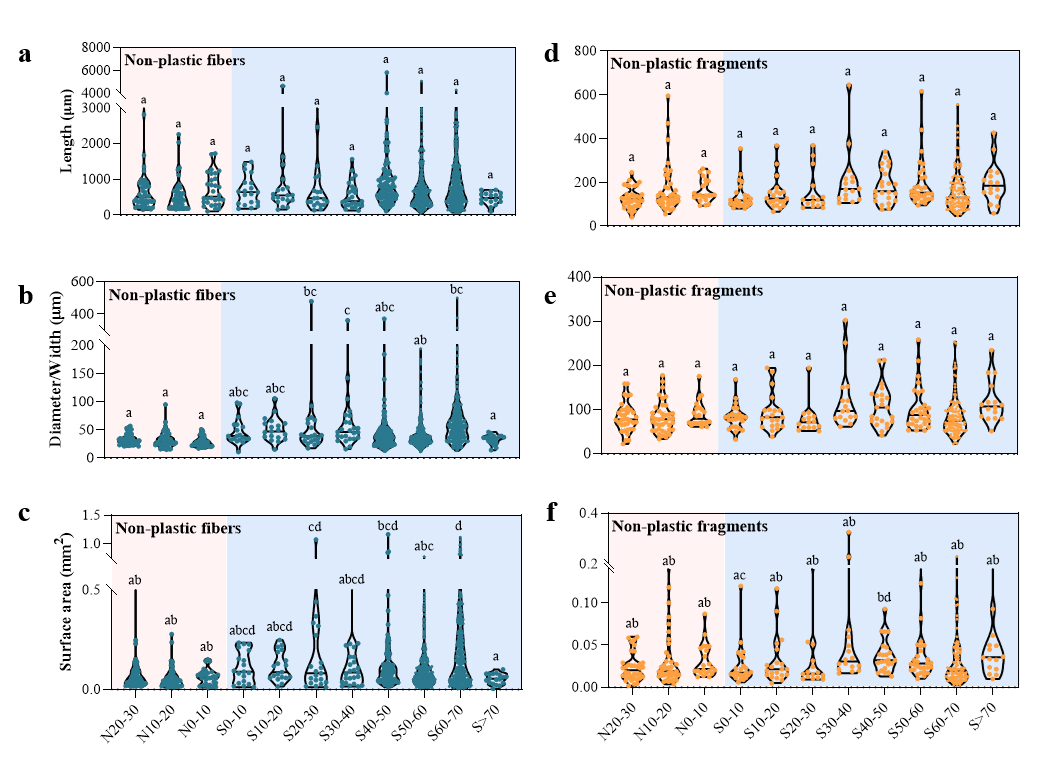


**Figure S12** Dimensional analysis of other non-polymer fibers and fragments. (a) fiber length; (b) fiber diameter; (c) fragment length; (d) fragment width. S indicates the latitude of the southern hemisphere, and S>70 indicates the regions in inland Antarctica. Red shaded area indicates the Northern Hemisphere, and blue shaded area indicates the Southern Hemisphere. The surface areas were calculated based on the particle dimensions and are not referring to the specific surface areas. Different letters in violin plots suggest significant differences according to ANOVA and LSD post hoc tests. Black lines that cross the violin plots are median values.

**Figure S13** Calculated mass values of microplastics (MPs) and other particles (i.e., fibers and fragments) along the cruise path in the Southern Hemisphere.


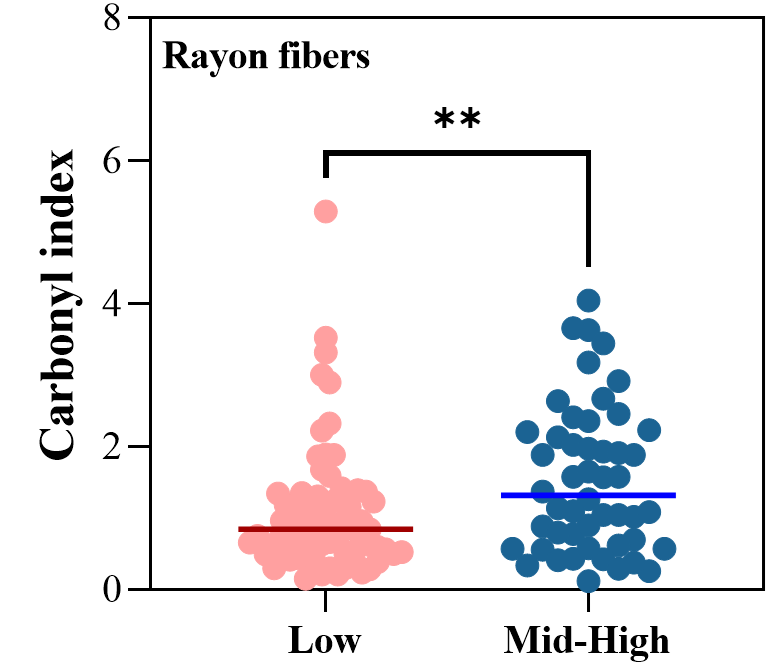


**Figure S14** The carbonyl index of the rayon fibers along latitudes. Low, Mid, and High latitudes represent the regions of approximately 30^o^N-30^o^S, 30-60^o^S, and 60-70^o^S along the cruise path, respectively. Lines in the middle indicate the mean values. The samples’ carbonyl index calculation was based on Eq 3, in which A_1_ area bands used for was of 1800-1670 cm^-1^, and A_2_ area bands used was of 1500-1390 cm^-1^, and when the A_1_ or A_2_ area values > 0.01, index was calculated. ** represents significant difference between the two groups (*p*<0.01).


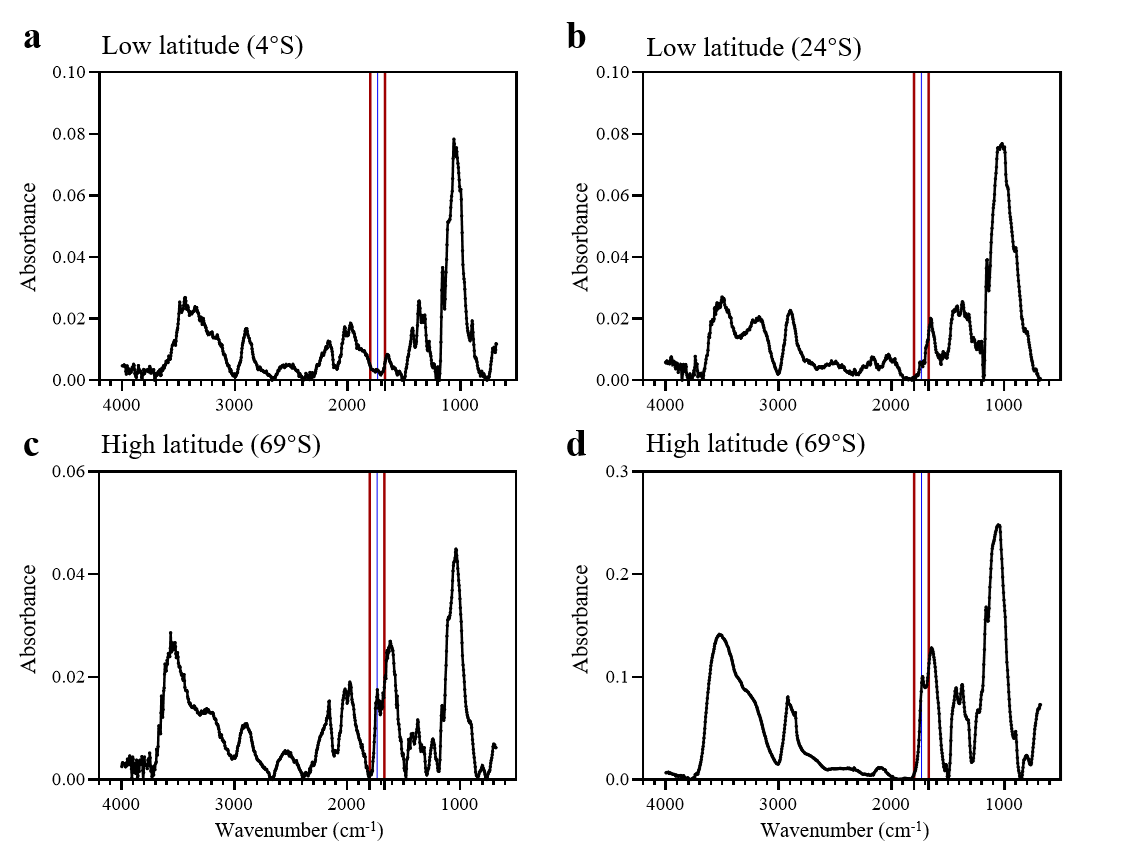


**Figure S15** Rayon micro-FTIR spectra comparison between samples collected from low (a-b) and high (c-d) latitudes. As the carbonyl index is a better indicator of weathering for polymers whose initial polymer structure does not contain C=O groups, and it would not linearly increase with latitudes during long-range transport, we chose to focus on rayon for a cross-latitude carbonyl index alteration analysis, which was frequently identified in samples collected. Furthermore, rayon does not contain C=O groups in pristine form. The C=O peak (at 1735 cm^-1^, blue lines) is more clearly shown for rayon collected at high latitudes, and the area under 1800 - 1670 cm^-1^ (red line intervals) was deemed as the absorbance area (A_1_) of the carbonyl (C=O) group, and the area under 1500 - 1390 cm^-1^ was deemed as the absorbance area (A_2_) of the reference (CH_2_) group in Eq 3.


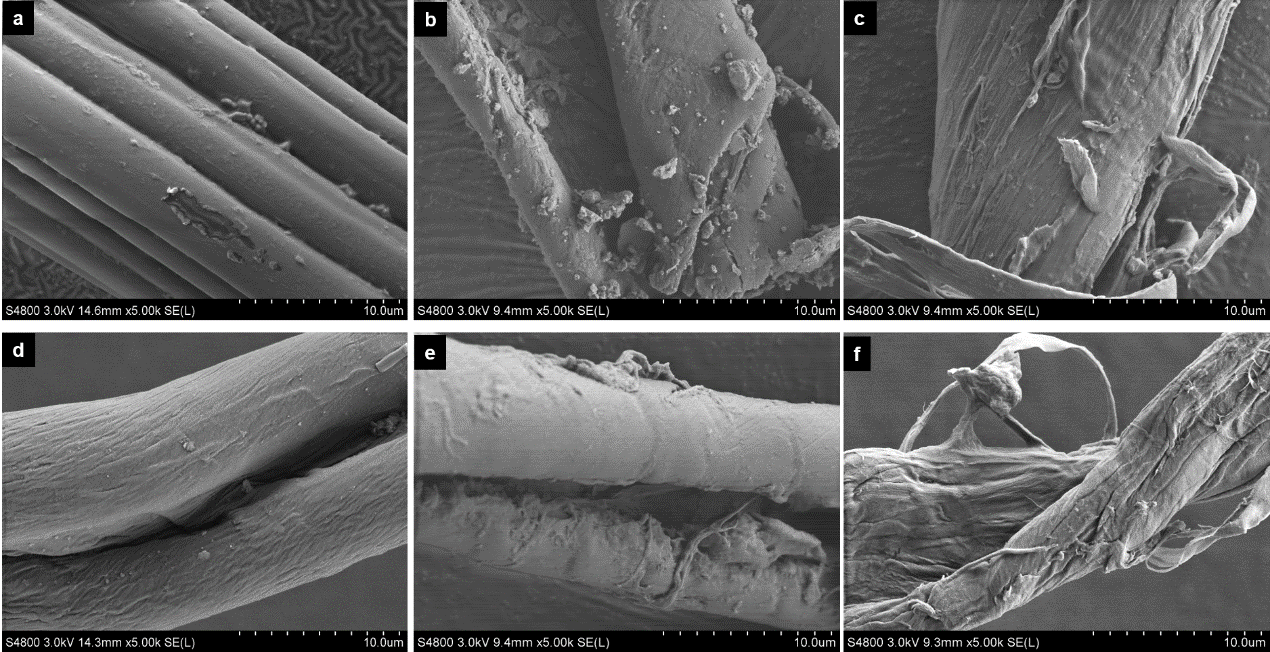


**Figure S16** Representative images of original compact fibers and loose fibers with voids collected from the air. (a) Original state of rayon fibers; (b-c) atmospheric rayon microfibers collected in Shanghai outdoor environment; (d) original state of cotton fibers; (e-f) atmospheric cotton microfibers collected in Shanghai outdoor environment. The images were re-edited from Supplementary Figures S3 and Figure S4 in Chen *et al. Environ* *Sci Eur* 2022, 34(1):25. Scanning electron microscope: S-4800, Hitachi, Japan; pictures were taken at 3.0 kV and 15 μA, and at a magnification of 5 K.


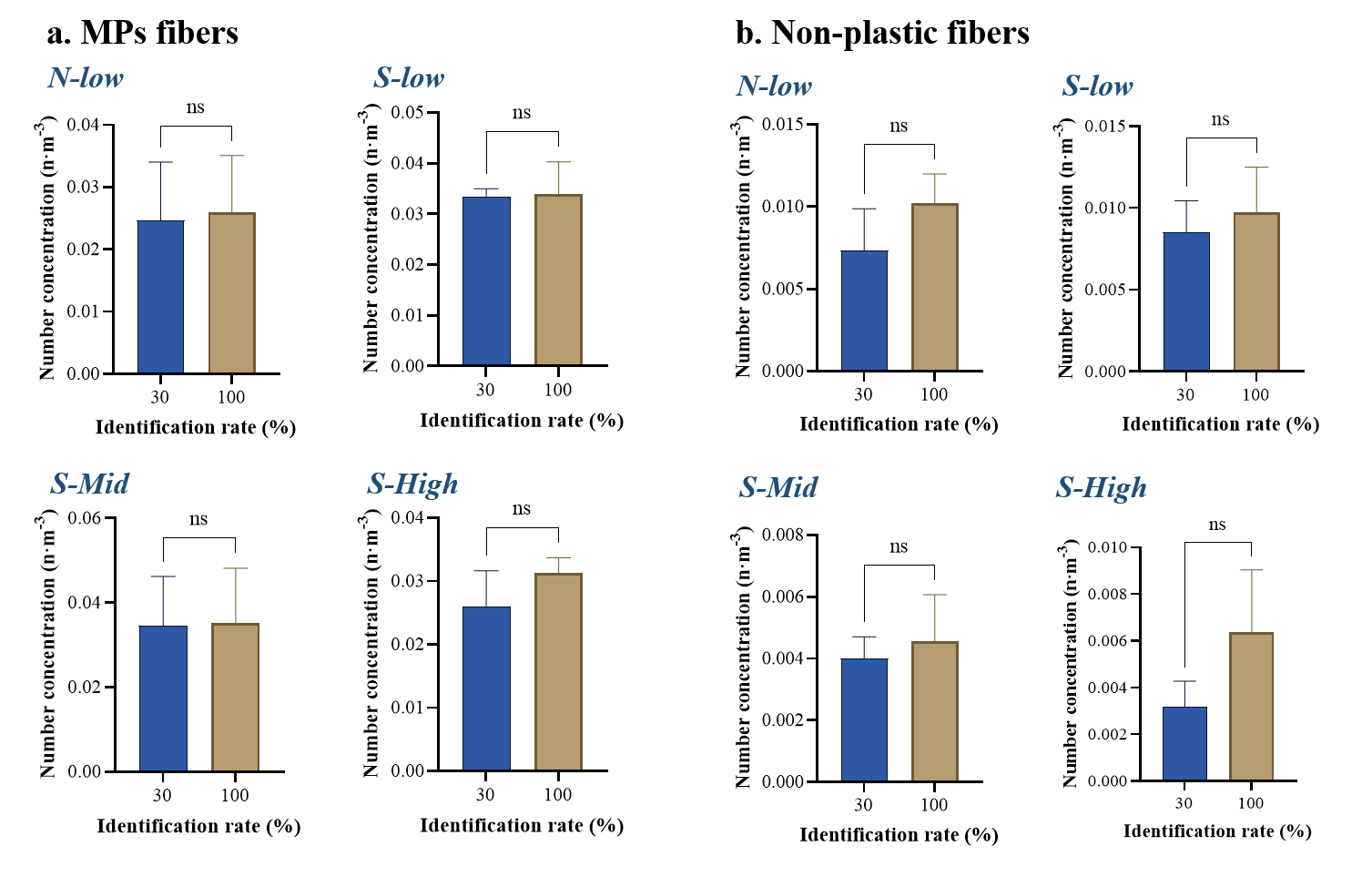


**Figure S17** Microplastics (MPs) and non-plastic particle concentrations comparison between the sub-sampling analysis (1^st^ analysis, 30% of fibers randomly selected and scaled) compared to measurement of all fibers in the sample (2^nd^ analysis, 100% particles quantified) for the 14 northerly samples across the campaign. (a) MPs fibers; (b) Non-plastic fibers. ns: indicates there was no statistically significant difference between the two groups (*p*>0.05) according to *t-*tests. Error bars represent one standard deviation.


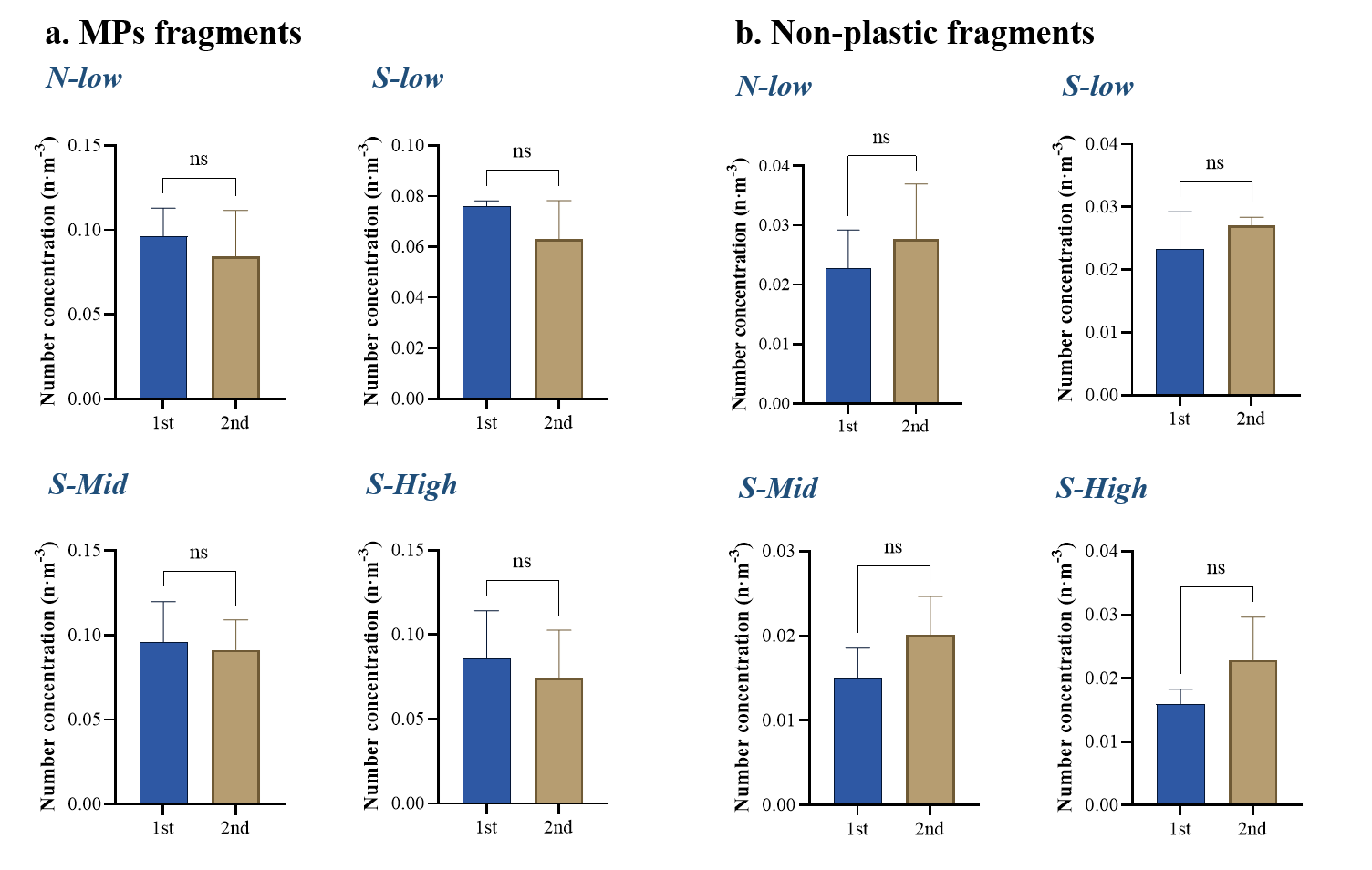


**Figure S18** Microplastics (MPs) and non-plastic fragments concentrations between the the1^st^ and the 2^nd^ one-fourth filters. (a) MPs fragments; (b) Non-plastic fragments. ns: indicates there is no significantly statistical difference between the two groups (*p*>0.05) according to *t-*tests. Error bars represent one standard deviation.

**Supplementary Tables**

**Table S1** Detailed information on the aerosol sampling in the marine boundary layer and inland Antarctica. Temperature of air was obtained from the shipboard automatic weather station.

| **No.** | **Sampling Period** | **Latitude**  **/^o^N^a^** | **Longitude**  **/^o^E ^a^** | **Sampling Volume/m^3^** | **Average Air Temperature/^o^C** | **Season** |
| --- | --- | --- | --- | --- | --- | --- |
| A01 | April 20-April 22, 2020 | 30.20 | 123.06 | 3168 | 15.1 | Spring |
| A02 | April 18-April 20, 2020 | 24.97 | 128.18 | 3581 | 20.3 | Spring |
| A03 | April 16-April 18, 2020 | 18.67 | 134.05 | 3802 | 26.5 | Spring |
| A04 | April 14-April 16, 2020 | 12.79 | 139.81 | 3008 | 27.9 | Spring |
| A05 | April 12-April 14, 2020 | 5.12 | 146.94 | 3851 | 28.8 | Spring |
| A06 | April 10-April 12, 2020 | -4.71 | 152.56 | 3433 | 29.2 | Autumn |
| A07 | April 8-April 10, 2020 | -13.66 | 155.27 | 3319 | 28.1 | Autumn |
| A08 | April 6-April 8, 2020 | -24.05 | 155.57 | 3583 | 25.1 | Autumn |
| A09 | April 4-April 6, 2020 | -34.27 | 153.05 | 3428 | 20.2 | Autumn |
| A10 | April 2-April 4, 2020 | -44.41 | 150.09 | 3553 | 13.3 | Autumn |
| A11 | November 7-November 10, 2019 | -45.85 | 147.42 | 4398 | 7.4 | Spring |
| A12 | November 10-November 12, 2019 | -54.24 | 144.16 | 3514 | 3.5 | Spring |
| A13 | November 12-November 14, 2019 | -60.34 | 129.58 | 3467 | -0.8 | Spring |
| A14 | November 14-November 16, 2019 | -60.89 | 109.60 | 3506 | -0.7 | Spring |
| A15 | November 16-November 18, 2019 | -61.65 | 89.95 | 3486 | -1.1 | Spring |
| A16 | March 14-March 16, 2020 | -64.04 | 103.43 | 3566 | -2.0 | Autumn |
| A17 | March 16-March 18, 2020 | -64.39 | 128.36 | 3376 | -2.4 | Autumn |
| A18 | March 18-March 20, 2020 | -64.74 | 146.33 | 3491 | -0.9 | Autumn |
| A19 | March 20-March 22, 2020 | -65.17 | 151.61 | 3406 | -0.7 | Autumn |
| A20 | March 22-March 24, 2020 | -65.27 | 152.93 | 3506 | -6.0 | Autumn |
| A21 | March 12-March 14, 2020 | -65.42 | 82.00 | 3364 | -6.7 | Autumn |
| A22 | November 18-November 20, 2019 | -65.62 | 77.77 | 3493 | -2.6 | Spring |
| A23 | November 20-November 22, 2019 | -68.97 | 76.42 | 3883 | -3.0 | Spring |
| A24 | November 22-November 25, 2019 | -69.30 | 76.23 | 4638 | -2.0 | Spring |
| A25 | January 21-January 25, 2020 | -73.86 | 76.97 | 6110 | -12.9 | Summer |
| A26 | January 26-January 30, 2020 | -73.86 | 76.97 | 5822 | -16.7 | Summer |

^a^ the mean values of individual sampling voyage legs.

**Table S2** Microplastics (MPs) and non-plastic particles number concentrations in the marine boundary layer and inland Antarctica.

| **Latitude**  **(^o^)** | **MPs Fibers**  **(n·m^-3^)** | **MPs Fragments**  **(n·m^-3^)** | **MPs Total**  **(n·m^-3^)** | **Non-plastic Fibers**  **(n·m^-3^)** | **Non-plastic Fragments**  **(n·m^-3^)** | **Non-plastic Particles Total**  **(n·m^-3^)** | **All Fibers**  **(n·m^-3^)** | **All Fragments**  **(n·m^-3^)** |
| --- | --- | --- | --- | --- | --- | --- | --- | --- |
| 30.20 | 0.0391 | 0.0088 | 0.0480 | 0.1136 | 0.0189 | 0.1326 | 0.1528 | 0.0278 |
| 24.97 | 0.0257 | 0.0123 | 0.0380 | 0.0760 | 0.0223 | 0.0983 | 0.1017 | 0.0346 |
| 18.67 | 0.0210 | 0.0105 | 0.0316 | 0.0431 | 0.0368 | 0.0800 | 0.0642 | 0.0473 |
| 12.79 | 0.0146 | 0.0080 | 0.0226 | 0.0864 | 0.0386 | 0.1250 | 0.1011 | 0.0465 |
| 5.12 | 0.0291 | 0.0114 | 0.0405 | 0.1028 | 0.0218 | 0.1246 | 0.1319 | 0.0332 |
| -4.71 | 0.0303 | 0.0105 | 0.0408 | 0.0699 | 0.0280 | 0.0979 | 0.1002 | 0.0384 |
| -13.66 | 0.0301 | 0.0121 | 0.0422 | 0.0458 | 0.0277 | 0.0735 | 0.0759 | 0.0398 |
| -24.05 | 0.0413 | 0.0067 | 0.0480 | 0.0737 | 0.0257 | 0.0994 | 0.1150 | 0.0324 |
| -34.27 | 0.0443 | 0.0035 | 0.0478 | 0.1038 | 0.0233 | 0.1272 | 0.1482 | 0.0268 |
| -44.41 | 0.0259 | 0.0056 | 0.0315 | 0.0788 | 0.0169 | 0.0957 | 0.1047 | 0.0225 |
| -45.85 | 0.0200 | 0.0045 | 0.0246 | 0.0755 | 0.0109 | 0.0864 | 0.0955 | 0.0155 |
| -54.24 | 0.0353 | 0.0046 | 0.0398 | 0.0774 | 0.0102 | 0.0877 | 0.1127 | 0.0148 |
| -60.34 | 0.0392 | 0.0058 | 0.0450 | 0.0715 | 0.0115 | 0.0831 | 0.1108 | 0.0173 |
| -60.89 | 0.0240 | 0.0057 | 0.0297 | 0.0958 | 0.0125 | 0.1084 | 0.1198 | 0.0183 |
| -61.65 | 0.0161 | 0.0034 | 0.0195 | 0.0654 | 0.0161 | 0.0815 | 0.0815 | 0.0195 |
| -64.04 | 0.0213 | 0.0034 | 0.0247 | 0.0718 | 0.0146 | 0.0864 | 0.0931 | 0.0179 |
| -64.39 | 0.0284 | 0.0059 | 0.0344 | 0.0675 | 0.0166 | 0.0841 | 0.0960 | 0.0225 |
| -64.74 | 0.0309 | 0.0046 | 0.0355 | 0.0378 | 0.0321 | 0.0699 | 0.0688 | 0.0367 |
| -65.17 | 0.0317 | 0.0047 | 0.0364 | 0.1045 | 0.0235 | 0.1280 | 0.1362 | 0.0282 |
| -65.27 | 0.0342 | 0.0103 | 0.0445 | 0.0867 | 0.0194 | 0.1061 | 0.1209 | 0.0297 |
| -65.42 | 0.0250 | 0.0036 | 0.0285 | 0.0832 | 0.0107 | 0.0939 | 0.1082 | 0.0143 |
| -65.62 | 0.0321 | 0.0092 | 0.0412 | 0.0618 | 0.0057 | 0.0676 | 0.0939 | 0.0149 |
| -68.97 | 0.0299 | 0.0041 | 0.0340 | 0.0690 | 0.0155 | 0.0845 | 0.0989 | 0.0196 |
| -69.30 | 0.0147 | 0.0078 | 0.0224 | 0.0612 | 0.0095 | 0.0707 | 0.0759 | 0.0172 |
| -73.86 | 0.0009 | 0.0018 | 0.0027 | 0.0071 | 0.0080 | 0.0151 | 0.0080 | 0.0098 |
| -73.86 | 0.0055 | 0.0028 | 0.0083 | 0.0092 | 0.0064 | 0.0156 | 0.0147 | 0.0092 |

**Table S3** Summary of airborne microplastics (MPs) concentrations in air samplers.

| **Microplastic air concentrations sampled over the ocean or coasts** | | | | | |
| --- | --- | --- | --- | --- | --- |
| **Location** | **Average microplastic counts^a^** | **Analysis method** | **Environment** | **Size range^b^** | **Reference** |
| From Pacific Ocean to Southern Ocean | 0.039, 0.032, 0.041, 0.048, 0.035, 0.038, 0.032 MP/m^3^ (offshore and pelagic areas) | µFTIR | Offshore and open ocean air | 20 µm -5 mm | This study |
| Atlantic coast, France | 2.9, 9.6 MP/m^3^ (on shore and off shore) | µRaman | Onshore and offshore air | 2.5 µm-300 µm | ^1^ |
| Atlantic Ocean | 0.0112 MP/m^3^ | µRaman | Offshore air | 5 µm-5 mm | ^2^ |
| South China Sea | 0.39 MP/100m^3^ | µFTIR | Offshore air | 20 µm -1 mm | ^3^ |
| Western Pacific Ocean | 0.13, 0.01 MP/m^3^ (coastal and pelagic areas) | µFTIR | Onshore and offshore air | 20 µm -2 mm | ^4^ |
| **Microplastic air concentrations sampled over the land** | | | | | |
| **Location** | **Average microplastic counts^a^** | **Analysis method** | **Environment** | **Size range^a^** | **Reference** |
| Safat, Kuwait | 8.9, 14.6, 4.15, 14.3 MP/m^3^ | µRaman | City air | 1 µm-5 mm | ^5^ |
| Weser River catchment, Germany | 121, 37, 115 MP/m^3^ | Raman | Rural and urban air | 4 µm-5 mm | ^6^ |
| Pic du Midi, France | 0.23 MP/m^3^ | µRaman | Rural air | 5 µm-163 µm | ^7^ |
|  |  |  |  |  |  |
| Shanghai, China | 1.42 MP/m^3^ | µFTIR | City air | 20 µm-9.55 mm | ^8^ |
| Cal State University, USA (outdoor) | 7.9 MP/m^3^ | µRaman, FTIR | City air | 20 µm ->3 mm | ^9^ |
| Madrid, Spain | 1.5, 3.2, 3.7, 13.9 MP/m^3^  (from rural to urban) | µFTIR | Rural and urban air | 25 µm-5 mm | ^10^ |

^a^ In some rows, multiple numbers representing the average concentrations of various sampling regions are presented for a single study.

^b^ References include microplastics studies but exclude nanoplastics studies.

**Table S4** The density values of dominant microplastics (MPs) and non-plastic particles in the atmospheric samples.

| **Categories** | **Airborne fibers^a^** | **Percentage** | **Density [kg·m^-3^]** | **Average Density [kg·m^-3^]** |
| --- | --- | --- | --- | --- |
| MPs fibers | Rayon | 13.79% | 1520 | 1471 |
|  | Polyester | 7.48% | 1380 |  |
| Non-plastic fibers | Cotton | 39.04% | 1550 | 1541 |
|  | Cellulose | 8.14% | 1500 |  |
|  |  |  |  |  |
| **Categories** | **Airborne fragments^a^** | **Percentage** | **Density [kg·m^-3^]** |  |
| MPs fragments | Rayon | 9.04% | 1520 | 1404 |
|  | Epoxy resin | 5.08% | 1200 |  |
| Non-plastic  fragments | Cotton | 3.11% | 1550 | 1525 |
|  | Cellulose | 3.11% | 1500 |  |

^a^ only the most dominant fibers and fragments components are shown.

**Table S5** Estimated microplastics (MPs) and non-plastic particles mass concentrations in the marine boundary layer and inland Antarctica.

| **Latitude**  **(^o^)** | **MPs Fibers**  **(μg·m^-3^)** | **MPs Fragments**  **(μg·m^-3^)** | **MPs total**  **(μg·m^-3^)** | **Non-plastic**  **Fibers**  **(μg·m^-3^)** | **Non-plastic**  **Fragments**  **(μg·m^-3^)** | **Non-plastic**  **Particles total**  **(μg·m^-3^)** | **All Fibers**  **(μg·m^-3^)** | **All Fragments**  **(μg·m^-3^)** |
| --- | --- | --- | --- | --- | --- | --- | --- | --- |
| 30.20 | 0.0407 | 0.0008 | 0.0415 | 0.0935 | 0.0063 | 0.0998 | 0.1342 | 0.0071 |
| 24.97 | 0.0276 | 0.0098 | 0.0374 | 0.0394 | 0.0263 | 0.0657 | 0.0670 | 0.0361 |
| 18.67 | 0.0093 | 0.0072 | 0.0165 | 0.0377 | 0.0170 | 0.0547 | 0.0470 | 0.0242 |
| 12.79 | 0.0029 | 0.0033 | 0.0061 | 0.0504 | 0.0188 | 0.0693 | 0.0533 | 0.0221 |
| 5.12 | 0.0062 | 0.0112 | 0.0175 | 0.0436 | 0.0202 | 0.0638 | 0.0498 | 0.0314 |
| -4.71 | 0.0279 | 0.0795 | 0.1073 | 0.1003 | 0.0204 | 0.1207 | 0.1282 | 0.0999 |
| -13.66 | 0.0378 | 0.0079 | 0.0457 | 0.1026 | 0.0323 | 0.1349 | 0.1404 | 0.0402 |
| -24.05 | 0.1792 | 0.0092 | 0.1885 | 0.4001 | 0.0133 | 0.4135 | 0.5794 | 0.0226 |
| -34.27 | 0.1910 | 0.0060 | 0.1970 | 0.2667 | 0.0675 | 0.3343 | 0.4577 | 0.0735 |
| -44.41 | 0.0120 | 0.0015 | 0.0135 | 0.0301 | 0.0094 | 0.0395 | 0.0421 | 0.0109 |
| -45.85 | 0.0253 | 0.0301 | 0.0554 | 0.2372 | 0.0271 | 0.2644 | 0.2626 | 0.0572 |
| -54.24 | 0.0644 | 0.0098 | 0.0743 | 0.1250 | 0.0106 | 0.1356 | 0.1895 | 0.0204 |
| -60.34 | 0.0448 | 0.0065 | 0.0513 | 0.0719 | 0.0167 | 0.0886 | 0.1167 | 0.0232 |
| -60.89 | 0.0467 | 0.0164 | 0.0632 | 0.1001 | 0.0344 | 0.1344 | 0.1468 | 0.0508 |
| -61.65 | 0.0127 | 0.0107 | 0.0233 | 0.0766 | 0.0247 | 0.1013 | 0.0892 | 0.0354 |
| -64.04 | 0.0184 | 0.0005 | 0.0190 | 0.0822 | 0.0038 | 0.0860 | 0.1007 | 0.0043 |
| -64.39 | 0.0567 | 0.0018 | 0.0585 | 0.2771 | 0.0045 | 0.2816 | 0.3338 | 0.0063 |
| -64.74 | 0.0078 | 0.0004 | 0.0081 | 0.0361 | 0.0035 | 0.0395 | 0.0438 | 0.0038 |
| -65.17 | 0.0086 | 0.0155 | 0.0241 | 0.2568 | 0.0234 | 0.2802 | 0.2654 | 0.0389 |
| -65.27 | 0.0143 | 0.0191 | 0.0334 | 0.3984 | 0.0461 | 0.4445 | 0.4127 | 0.0652 |
| -65.42 | 0.0463 | 0.0029 | 0.0491 | 0.2068 | 0.0055 | 0.2123 | 0.2530 | 0.0084 |
| -65.62 | 0.1711 | 0.0043 | 0.1754 | 0.3926 | 0.0117 | 0.4042 | 0.5637 | 0.0159 |
| -68.97 | 0.2205 | 0.0033 | 0.2238 | 0.3895 | 0.0083 | 0.3978 | 0.6099 | 0.0116 |
| -69.30 | 0.1396 | 0.0033 | 0.1429 | 0.3029 | 0.0085 | 0.3114 | 0.4425 | 0.0118 |
| -73.86 | 0.0002 | 0.0005 | 0.0008 | 0.0020 | 0.0132 | 0.0152 | 0.0022 | 0.0138 |
| -73.86 | 0.0029 | 0.0032 | 0.0061 | 0.0050 | 0.0206 | 0.0257 | 0.0080 | 0.0238 |

**Table S6** Comparison of microplastics (MPs) and non-plastic fibers concentrations between an entire analysis (100% particles were fully scanned) and a sub-sampling analysis (30% randomly selected particles on the filter) for 14 samples.

| **Latitude** | **MPs Fibers^a^** | **MPs Fibers^b^** | **Non-plastic Fibers^a^** | **Non-plastic Fibers^b^** |
| --- | --- | --- | --- | --- |
| **(^o^)** | **(n·m^-3^)** | **(n·m^-3^)** | **(n·m^-3^)** | **(n·m^-3^)** |
| -64.39 | 0.0237 | 0.0284 | 0.0671 | 0.0675 |
| -64.74 | 0.0344 | 0.0309 | 0.0573 | 0.0378 |
| -65.17 | 0.0235 | 0.0317 | 0.1135 | 0.1045 |
| -65.27 | 0.0228 | 0.0342 | 0.1065 | 0.0867 |
| -44.41 | 0.0263 | 0.0259 | 0.0788 | 0.0788 |
| -34.27 | 0.0428 | 0.0443 | 0.1128 | 0.1038 |
| -24.05 | 0.0335 | 0.0413 | 0.0781 | 0.0737 |
| -13.66 | 0.0321 | 0.0301 | 0.0763 | 0.0458 |
| -4.71 | 0.035 | 0.0303 | 0.0738 | 0.0699 |
| 5.12 | 0.0277 | 0.0291 | 0.1039 | 0.1028 |
| 12.79 | 0.0177 | 0.0146 | 0.0886 | 0.0864 |
| 18.67 | 0.014 | 0.0210 | 0.0842 | 0.0431 |
| 24.97 | 0.0261 | 0.0257 | 0.0819 | 0.0760 |
| 30.20 | 0.0379 | 0.0391 | 0.1221 | 0.1136 |

**^a^** indicates data where 30% of fibers were measured on ¼ filter and scaled, shown in black values.

**^b^** indicates data where 100% of fibers were measured on ¼ filter, shown in blue values.

**Table S7** Comparison of microplastics (MPs) and non-plastic fragments concentrations between the 1^st^ and the 2^nd^ analysis. We made replicate analysis on two different ¼ of the whole filter, and the fragments concentrations results do not show statistically significant differences according to *t*-tests.

| **Latitude** | **MPs Fragments^a^** | **MPs Fragments^b^** |
| --- | --- | --- |
| **(^o^)** | **(n·m^-3^)** | **(n·m^-3^)** |
| -64.39 | 0.0024 | 0.0059 |
| -64.74 | 0.0023 | 0.0046 |
| -65.17 | 0.0035 | 0.0047 |
| -65.27 | 0.0046 | 0.0103 |
| -44.41 | 0.0045 | 0.0056 |
| -34.27 | 0.0035 | 0.0035 |
| -24.05 | 0.0067 | 0.0067 |
| -13.66 | 0.0084 | 0.0121 |
| -4.71 | 0.0105 | 0.0105 |
| 5.12 | 0.0083 | 0.0114 |
| 12.79 | 0.0106 | 0.0080 |
| 18.67 | 0.0063 | 0.0105 |
| 24.97 | 0.0078 | 0.0123 |
| 30.20 | 0.0038 | 0.0088 |

**^a^** results where 30% of fibers were measured on ¼ filter and scaled, shown in black values.

**^b^** results where 100% of fibers were measured on ¼ filter, shown in blue values.

**Supplementary Texts**

**Text S1 The linear regression hypothesis background**

Hicks et al. found that the dry settling velocity of atmospheric aerosol particles is directly proportional to friction velocity ^11^; and Vong et al. further obtained the formula of dry settling velocity after dimensionless treatment with friction velocity ^12^.

*V_d_* = *C* (*U_f_* ) *D* [1+(−300/*L*)^2/3^] (S1)

*V_d_* is the settling velocity and has a unit of cm s^−1^; *C* is a numerical coefficient; *U_f_* is the friction velocity and has units of m s^−1^; *L* is the Monin–Obukhov length and has units of m; and *D* is the diameter of the particle and has units of µm.

Therefore, we assume for a specific-sized particle, its *V_d_* is close to a constant. Thus, we hypothesize that the relationship between distance and MPs concentrations follows the linear relationship.

**Text S2 Atmospheric particles concentrations comparison between the sub-sampling and full analysis**

Atmospheric particles were collected onto Whatman quartz fiber filters (20.3 cm × 25.4 cm) at each site. Typically, for a sampling duration of 48 h, this would consequently lead to sampling a volume of 3456 m^3^ on each filter. For sample analysis, one fourth of each filter was used for particles characterization and quantification according to a previous airborne particulate study^13^. Two morphologies were included here, namely fibers and fragments, and two rounds of analysis were conducted, as detailed below.

***1^st^ analysis:***

As the fibers number concentrations decreased for more southerly latitudes, we found that 14 samples in the more northerly sample locations had too many fibers on one fourth of the filters to easily measure (number of fibers exceeded one hundred). To facilitate the identification and quantitation, all fibers were observed and collected under the microscope and the total numbers were counted. Then, we randomly selected 30% of the fibers to identify their composition chemistries. Therefore, of the 26 sampling sites, we identified all fibers for 12 sampling sites (i.e., southern sites) and 30% of the fibers for 14 sampling sites (i.e., northern sites), and adjusted the final fiber number concentrations and chemical identification reported accordingly during the 1^st^ analysis. Fragments in all 26 samples were counted and chemical compositions were identified across the entire expedition.

***2^nd^ analysis:***

For method verification, we used the backup samples (another one fourth of the filters) during the 2^nd^ analysis for a full scan to prove the robustness of this sub-sampling approach (i.e., only using 30% of the fibers in the 1^st^ analysis) by comparing these values with the entire analysis (100% of fibers analyzed in the 2^nd^ analysis). The impacts of sub-sampling a filter versus identifying the entire sample did not have large discrepancies in this instance (Table S6, Figure S17). Here, the fiber concentrations obtained between sub-sampling and the full analysis were similar, when scaled for the proportion of the filter which was analyzed. Beyond our specific study, this may be useful for other researchers in the future to assess whether a full scan needs to be performed or only a sub-section analysis is sufficient if situations where a large number of microplastics are recovered. However, we appreciate that by nature microplastics contamination can be heterogeneous, and so an assessment of the goodness of fit of subsampling should be considered in any study which does not measure the entire particle distribution in a sample.

During both the 1^st^ and the 2^nd^ analysis, all fragments (100%) on filters were identified, and a comparison of the fragments concentrations indicates that there is no significant difference between the two measurements for MPs fragments and non-plastic fragments according to *t-*tests (Table S7, Figure S18).

**Text S3 Mass and surface area estimation for fiber and fragment particles**

We used the cylinder model ^14^ and the column model ^15^ to estimate the surface area and volume (mass) values of fibers and fragments. Additionally, an approximation by assuming the length-to-width ratio equates to the width-to-height ratio (*L*/*W*=*W*/*H*) was also applied for fragments ^14, 16^.

For the microplastic fibers, we measured the projected width and length of particles utilizing ImageJ. The width was equivalent to the diameter of the bottom surface of a cylinder, the length was equivalent to the length of the cylinder, and calculated the mass according to Equation 1. For the microplastic fragments, we measured the projected length and width, utilizing ImageJ directly, projected the height using *L*/*W*=*W*/*H*, and calculated the mass according to Equation 2.

MC_fiber_ =∑n k=1(1-*f*)·(*R_k_*^2^·*L_k_*) π*ρ*/*v*_i_ (1)

MC_fragment_=∑n k=1 *S_k_·(W_k_^2^/L_k_) ρ/v_i_* (2)

we approximated fibers to be cylinders and considered a void fraction ( *f* =40%)^14^, and *n* is in the total number of fibers in the sample, *i*; v_i_ is the sampled air volume. *R* and *L* were the fiber diameter and length as calculated by the ImageJ software according to the top-view projection images, respectively. *ρ* is the average density of primary fibers collected in this survey (Table S4). For instance, rayon and polyester were the dominant MPs fibers detected, and rayon and epoxy resin were the dominant MPs fragments detected. Therefore, the average densities for MPs fibers and MPs fragments were calculated based on these primary MPs densities and their composition percentages of 1471 kg·m^-3^ and 1404 kg·m^-3^, respectively. Further, we estimated the surface area (SA) of each particle (Figures 4, Figure S11).

SA_fiber_ = $2\pi R\cdot L+\pi R^{2}/2$ (S2)

SA_fragment_ =4$LW+{2W}^{2}$ (S3)

Collectively, after obtaining the diameter, width, and length parameters, we estimated the surface area, volume (mass) of each particle. However, it is important to note that this simplified approach to suggest surface area and volume (mass) likely represents a low estimate, as it does not take into account additional surface roughness and cracks which are likely to be present on environmental microplastics, subsequently increasing the actual surface area.

**Text S4 Carbonyl index calculation**

It is true that the carbonyl index will not linearly increase with latitudes during long-range transport. Therefore, when analyzing environmental samples, a large sample size is important to indicate a general trend of the carbonyl index. Moreover, the carbonyl index is a better indicator of weathering for polymers whose initial polymer structure does not contain C=O groups. For instance, polyester contains carbonyl groups and consequently any newly formed carbonyl signals attributed to polymer aging may not be easily distinguishable from the background^17^. Therefore, the carbonyl index alteration has not been observed for polyester fibers after UV weathering^18^. For the two reasons mentioned above, we chose to focus on rayon for a cross-latitude carbonyl index alteration analysis, which has a large sample number and the material does not contain C=O in its pristine form. We have further supplemented a diagram of rayon’s spectra comparison between low and high latitudes (Figure S15), where the C=O peak (at 1735 cm^-1^, blue lines) was more clearly shown for rayons collected at higher latitudes, and the area under1800 - 1670 cm^-1^ (red line intervals) was deemed as the absorbance area (A_1_) of the carbonyl (C=O) group , and the area under 1500 - 1390 cm^-1^ was deemed as the absorbance area (A_2_) of the reference (CH_2_) group in Equation 3.

CI=*A_1_*/*A_2_* (3)

where, A_1_ is the absorbance of the carbonyl (C=O) peak for MPs, and A_2_ is the absorbance of a reference peak (CH_2_) for these MPs samples.

**References cited in the Supporting Information:**

1. Allen S, Allen D, Moss K, Le Roux G, Phoenix VR, Sonke JE. Examination of the ocean as a source for atmospheric microplastics. *PloS one* 2020, **15**(5)**:** e0232746.

2. Trainic M, Flores JM, Pinkas I, Pedrotti ML, Lombard F, Bourdin G*, et al.* Airborne microplastic particles detected in the remote marine atmosphere. *Commun Earth Environ* 2020, **1**(1)**:** 64.

3. Wang X, Liu K, Zhu L, Li C, Song Z, Li D. Efficient transport of atmospheric microplastics onto the continent via the East Asian summer monsoon. *J Hazard Mater* 2021, **414:** 125477.

4. Liu K, Wu T, Wang X, Song Z, Zong C, Wei N*, et al.* Consistent transport of terrestrial microplastics to the ocean through atmosphere. *Environ Sci Technol* 2019, **53**(18)**:** 10612-10619.

5. Uddin S, Fowler SW, Habibi N, Sajid S, Dupont S, Behbehani M. A preliminary assessment of size-fractionated microplastics in indoor aerosol—Kuwait’s baseline. *Toxics* 2022, **10**(2)**:** 71.

6. Kernchen S, Löder MG, Fischer F, Fischer D, Moses SR, Georgi C*, et al.* Airborne microplastic concentrations and deposition across the Weser River catchment. *Sci Total Environ* 2022, **818:** 151812.

7. Allen S, Allen D, Baladima F, Phoenix V, Thomas J, Le Roux G*, et al.* Evidence of free tropospheric and long-range transport of microplastic at Pic du Midi Observatory. *Nat Commun* 2021, **12**(1)**:** 1-10.

8. Liu K, Wang X, Fang T, Xu P, Zhu L, Li D. Source and potential risk assessment of suspended atmospheric microplastics in Shanghai. *Sci Total Environ* 2019, **675:** 462-471.

9. Zhang K, Su J, Xiong X, Wu X, Wu C, Liu J. Microplastics differ between indoor and outdoor air masses: insights from multiple microscopy methodologies. *Appl spectrosc* 2020, **74**(9)**:** 1079-1098.

10. González-Pleiter M, Edo C, Aguilera Á, Viúdez-Moreiras D, Pulido-Reyes G, González-Toril E*, et al.* Occurrence and transport of microplastics sampled within and above the planetary boundary layer. *Sci Total Environ* 2021, **761:** 143213.

11. Hicks BB, Saylor RD, Baker BD. Dry deposition of particles to canopies—A look back and the road forward. *J Geophys Res Atmos* 2016, **121**(24)**:** 14-691.

12. Vong RJ, Vong IJ, Vickers D, Covert DS. Size-dependent aerosol deposition velocities during BEARPEX'07. *Atmospheric Chem Phys* 2010, **10**(12)**:** 5749-5758.

13. Moch JM, Dovrou E, Mickley LJ, Keutsch FN, Liu Z, Wang Y*, et al.* Global importance of hydroxymethanesulfonate in ambient particulate matter: Implications for air quality. *J Geophys Res-Atmos* 2020, **125**(18)**:** e2020JD032706.

14. Simon M, van Alst N, Vollertsen J. Quantification of microplastic mass and removal rates at wastewater treatment plants applying Focal Plane Array (FPA)-based Fourier Transform Infrared (FT-IR) imaging. *Water Res* 2018, **142:** 1-9.

15. Koelmans AA, Redondo-Hasselerharm PE, Mohamed Nor NH, Kooi M. Solving the nonalignment of methods and approaches used in microplastic research to consistently characterize risk. *Environ Sci Technol* 2020, **54**(19)**:** 12307-12315.

16. Mintenig SM, Kooi M, Erich MW, Primpke S, Redondo-Hasselerharm PE, Dekker SC*, et al.* A systems approach to understand microplastic occurrence and variability in Dutch riverine surface waters. *Water Res* 2020, **176:** 115723.

17. Li J, Wang L, Xu Z, Zhang J, Li J, Lu X*, et al.* A new point to correlate the multi-dimensional assessment for the aging process of microfibers. *Water Res* 2023, **235:** 119933.

18. Pinlova B, Nowack B. Characterization of fiber fragments released from polyester textiles during UV weathering. *Environ Pollut* 2023, **322:** 121012.
